# Is There Any Relationship between Upper and Lower Limb Impairments in People with Multiple Sclerosis? A Kinematic Quantitative Analysis

**DOI:** 10.1155/2019/9149201

**Published:** 2019-10-09

**Authors:** Giancarlo Coghe, Federica Corona, Giuseppina Pilloni, Micaela Porta, Jessica Frau, Lorena Lorefice, Giuseppe Fenu, Eleonora Cocco, Massimiliano Pau

**Affiliations:** ^1^Department of Medical Sciences and Public Health, University of Cagliari, Cagliari, Italy; ^2^Department of Mechanical, Chemical and Materials Engineering, University of Cagliari, Cagliari, Italy

## Abstract

**Background:**

In people with multiple sclerosis (pwMS), disability is generally assessed on the basis of ambulation abilities, whereas upper limb motor dysfunctions are less frequently considered. Nevertheless, they can severely affect the quality of life of pwMS. To date, it remains mostly unknown whether a relationship exists between upper and lower limb impairments.

**Aim:**

To investigate the existence of a relationship between upper and lower limb impairments in pwMS based on two fundamental motor tasks, namely walking and hand-to-mouth (HTM) movement.

**Methods:**

Twenty-eight pwMS with Expanded Disability Status Scale (EDSS) scores in the range of 1–6, and 21 healthy controls (HC) underwent a kinematic analysis of gait and HTM movement performed with a motion capture system. The spatiotemporal parameters for the two tasks were calculated and correlated using Spearman's rank correlation coefficients.

**Results:**

The pwMS performed worse than HC on both tasks. Small to large correlations were found between the total HTM movement duration and most of the gait parameters (rho, 0.35–0.68; *p* < 0.05).

**Conclusions:**

Both upper and lower limb motor abilities in pwMS worsen as disability increases. Nevertheless, their relationship is only moderate. This finding emphasizes the need for specific tests to quantify disability considering the overall motor function in pwMS.

## 1. Introduction

In people with multiple sclerosis (pwMS), motor dysfunctions like ataxia, spasticity, incoordination, and sensory disturbances can affect the functionality of both lower and upper limbs. In the former case, they are likely to be responsible for walking abnormalities, usually expressed in the form of increased double support time and reduced gait speed, step length, and cadence [1], which are very common in pwMS even in the early stages of the disease [2]. Nevertheless, approximately 50% of these patients also complain of upper limb impairments such as muscle weakness, tremor, and clumsiness [3–5]. These symptoms may severely affect most activities of daily living and may worsen as the disease progresses [6], reducing the patients' degree of community participation and overall quality of life [7, 8].

It is noteworthy that the most frequently used clinical tool to assess the level of disability in MS (i.e., the Expanded Disability Status Scale, EDSS [9]) is essentially based on walking abilities, with the underlying assumption being that they are the main factor affected by the disease [9, 10]. While this approach is certainly justified by the higher frequency and severity of lower limb dysfunction in pwMS since longer neural pathways are more likely to be involved in central nervous system damage (e.g., cerebellar demyelination [11]), according to this framework upper limb impairment is neglected or, at the very least, is implicitly considered to have a similar impact as walking impairments on overall disability. However, this might represent a serious drawback if upper and lower limb impairments were poorly or not correlated. Unfortunately, to our knowledge, such data are not available. Clinicians have also available a range of tools specifically devoted to assess upper limb functions in MS. Among them, the most frequently employed are the Nine-Hole Peg Test, the Box and Block Test, and the Action Research Arm Test [3]), even though they are task specific and certainly less common than the EDSS in daily clinical practice [3, 12].

A detailed assessment of the functional impairments in pwMS can be obtained using quantitative 3-dimensional (3D) motion analysis, which is considered the gold standard for human movement evaluation. In pwMS, this approach has been extensively used to assess gait deterioration (for a detailed review, see [13]), while there are only few studies which used such tool to analyse the possible alterations in upper limb kinematics as a result of MS during the execution of functional tasks [14–16]. However, it was found that quantitative kinematic analysis is able to characterize the upper limb impairments during the execution of functional tasks with respect to unaffected individuals and several kinematic variables of interest are significantly correlated with the level of disability, dexterity, and muscular strength [16].

It is noteworthy that, to our knowledge, no study has investigated the existence of possible relationships between alterations in upper and lower limb kinematics. A detailed quantitative assessment of both types of impairments in the same individual would be valuable in clarifying the nature of differences in the impact of the disease on the two districts, and thereby establish whether it is necessary (and in what cases) to perform distinct assessments. On the basis of the above-mentioned considerations, the objectives of the present study were: (1) to quantitatively explore the relationship between lower and upper limb impairments on two common functional motor tasks, namely walking and hand-to-mouth movement; (2) to understand if such relationships are similar to those in unaffected individuals; and (3) to verify the types of differences that exist (if any) in the correlations between EDSS scale and the kinematic variables identified as representative of the investigated motor tasks, respectively, for upper and lower limbs. The results of the study should help further clarify if walking assessment alone is sufficient to fully represent the overall motor disabilities in MS, or if complementary measurements specific for upper limb evaluations should be performed.

## 2. Methods

### 2.1. Participants

A convenience sample of 28 pwMS (14 men, 14 women; mean age, 45.3 ± 9.7 years, mean disease duration: 18.5 ± 4.8 years) with an EDSS score in the range of 1–6 (mean, 4.0 ± 1.8, mean pyramidal subscore 4.7 ± 1.9, mean cerebellar subscore 2.0 ± 2.2, mean sensory subscore 3.0 ± 1.5) followed at the Regional Center for Multiple Sclerosis of Sardinia (Italy) was enrolled for the study. Twenty-five participants were characterized by paraparetic phenotype during the disease course, while 3 exhibit a prominent emiparetic involvement. The main inclusion criteria were a diagnosis of MS according to the 2005 McDonald criteria [17], absence of relapses in the 3 months before the study, ability to walk independently for at least 10 m, and absence of orthopaedic or cognitive disorders that could potentially interfere with the measurement protocol. The disability of each participant was evaluated by neurologists with expertise in MS (GC, EC, JF, LL and GF). An equally sized group of age-matched healthy individuals, recruited among pwMS caregivers, spouses, and hospital staff, formed the control group (HC). The main anthropometric and clinical characteristics of the participants are reported in Table 1.

The study was approved by the local ethics committee, and all participants signed a written informed consent form with detailed information about the aims of the study.

### 2.2. Experimental Procedure

All participants underwent kinematic analysis of two tasks:

1. Gait analysis (for the lower limbs).
2. Hand-to-mouth task (for the upper limbs). This task was selected as it was representative of common activities of daily living (like drinking and feeding) and has been already investigated in previous studies that aimed to characterize upper limb impairments in pwMS [16, 18].

In both cases, a motion capture system equipped with 8 infrared cameras set at a frequency of 120 Hz (BTS SMART-D; BTS Bioengineering, Milan, Italy) was employed. For each test, at least 6 trials were performed.

The gait analysis was preceded by acquisition of anthropometric measures (i.e., height, body mass, anterior superior iliac spine breadth, pelvis depth, knee and ankle width, and leg length) and placement of 22 spherical retro-reflective markers (14 mm in diameter) on the trunk and lower limbs at specific landmarks, according to the protocol described by Davis et al. [19]. The participants were required to walk barefoot along a 10 m walkway at a comfortable self-selected speed, ensuring suitable resting times between the trials.

For the hand-to-mouth task, 19 markers were positioned on the participant's head, trunk, and upper limbs, as described by Rab et al. [20]. The participants comfortably sat on a chair positioned in front of a table on which they were instructed to place their hands palms down, while the shoulders and the wrists assumed a neutral position, with the elbows flexed at approximately 90° and the forearm prone [18, 21–23]. Then, they were asked to perform the movement at a self-selected pace, as follows: from the starting position, they moved their hand to touch their mouth with the fingertips and then returned it to the starting position. The mean value of each kinematic parameter, calculated on the basis of three trials per limb, was considered representative of a certain participant and used for the subsequent analysis.

### 2.3. Kinematic Data Extraction

The raw data (i.e., 3D trajectories of the markers) were processed with dedicated software (Smart-Analyzer; BTS Bioengineering, Milan, Italy) to compute the set of kinematic parameters of interest. In particular, speed, stride length, cadence, and step width were calculated for gait. In contrast, hand-to-mouth movement was segmented into the following three phases, as described in previous studies [15, 23, 24]:

1. Going phase (GP), which involves the hand movement from the table to the mouth;
2. Adjusting phase (AP), which is dedicated to precisely locating the mouth;
3. Returning phase (RP), which refers to the movement of the hand back to the initial position.

By processing the marker trajectories, the following parameters were computed:

- Durations of the GP, AP, and RP (expressed as a percentage of the total time);
- Mean velocity of the hand markers during the GP;
- Adjustment sway (AS, mm), which is a measure of movement precision expressed as the overall length of the fingertip trajectory during the AP [15, 25].

### 2.4. Statistical Analysis

A preliminary analysis was performed to assess the existence of possible differences in the investigated parameters between the left and right limbs. Since no such differences were found, the mean value of each investigated parameter calculated across the two limbs was considered representative of each participant.

The possible differences introduced in both gait and the hand-to-mouth task by the presence of the disease were explored using a one-way multivariate analysis of variance (MANOVA) considering the participant's status (pwMS or HC) as independent variables and the 4 (gait), or 5 (hand-to-mouth) previously listed spatiotemporal parameters as dependent variables. The level of significance was set at *p* = 0.05, and the effect sizes were assessed using the eta-squared (*η*^2^) coefficient. Univariate ANOVA was carried out as a post-hoc test by reducing the level of significance to *p* = 0.0125 (0.05/4) for gait and *p* = 0.01 (0.05/5) for hand-to-mouth parameters after a Bonferroni correction for multiple comparisons.

The relationship between gait and hand-to-mouth parameters was assessed using Spearman's rank correlation analysis by setting the level of significance at *p* = 0.05. A similar approach was also used to establish the correlation between the EDSS score and the values of the investigated kinematic parameters. Rho values of 0.1, 0.3, and 0.5 were assumed to be representative of small, moderate, and large correlations, respectively [26]. All analyses were performed using SPSS Statistics v.20 (IBM, Armonk, NY, USA).

## 3. Results

The kinematic parameters calculated for the gait and hand-to-mouth task in pwMS and unaffected individuals are reported in Table 2, while Table 3 provides Spearman's rho coefficients for the correlation analysis between the two investigated tasks. The results of the correlation analysis between the EDSS score and the kinematic variables of the two tasks are presented in Table 4.

MANOVA revealed a significant effect of individual status on gait parameters [*F*(4,85) = 22.07, *p* < 0.001, Wilks *λ* = 0.49, *η*^2^ = 0.51]. In particular, the follow-up ANOVA showed that pwMS exhibit significantly reduced gait velocity (0.74 vs. 1.28 m/s in unaffected participants, *p* < 0.001), stride length (0.94 vs. 1.36 m, *p* < 0.001), and cadence (90.9 vs. 115.2 steps/min, *p* < 0.001) and increased step width (0.22 vs. 0.19 m, *p* = 0.001).

Similarly, a significant main effect of status was found on the hand-to-mouth parameters [*F*(5,84) = 15.22, *p* < 0.001, Wilks *λ* = 0.52, *η*^2^ = 0.47]. The subsequent ANOVA showed that pwMS are characterized by reduced velocity of the movement (0.51 vs. 0.58 m/s, *p* < 0.001) and altered magnitude of the three phases in which the movement was segmented. In fact, pwMS spent a significantly larger amount of time in the AP (7.1% vs. 3.7% of unaffected individuals, *p* < 0.001), exhibiting a shorter GP and longer RP.

The correlation analysis between upper and lower limb parameters in the two groups showed that, with respect to the investigated 20 correlations, pwMS exhibited 11 significant moderate to large correlations, while unaffected individuals showed only five significant correlations. In particular, in pwMS, gait speed was correlated with all hand-to-mouth parameters, with the exception of the GP duration. Moreover, the adjusting sway was found to be significantly correlated with all gait parameters, with the exception of the step width. In unaffected individuals, three hand-to-mouth variables (i.e., movement velocity, AP duration, and adjusting sway) were significantly correlated with gait speed.

The overall disability, expressed by the EDSS score, was found to be significantly correlated with all gait parameters with large and negative rho values in case of gait speed (−0.819, *p* < 0.001), stride length (−0.810, *p* < 0.001), and cadence (−0.745, *p* < 0.001) and positive moderate values for step width (0.389, *p* < 0.001). For the hand-to-mouth movement, all parameters except duration of the RP were found to be significantly correlated with EDSS. Positive moderate to large values were found for the AP duration (0.422, *p* < 0.001) and adjusting sway (0.275, *p* < 0.001), while negative values were observed for the mean velocity of the movement (−0.353, *p* < 0.001) and duration of the GP (0.427, *p* < 0.001).

## 4. Discussion

The main purpose of the present study was to verify the existence of a relationship between functional impairments of upper and lower limb in pwMS on the basis of two motor tasks (i.e., gait, and hand-to-mouth) representative of common activities of daily living. The major novel aspect of the study is characterized by the multiple quantitative kinematic analyes performed on the same individual to simultaneously characterize upper and lower limb impairments.

First, it is noteworthy that, as expected, pwMS performed worse than unaffected individuals in both investigated tasks. In this context, our results confirm the results of most previous studies on gait (see the review [27] for details) and are consistent with both the limited existing data on the hand-to-mouth task [14–16, 28] as well as those reported for similar upper limb precision tasks [29]. Moreover, the relationship between the kinematic parameters of gait and hand-to-mouth task also seems to be more evident in pwMS with respect to unaffected individuals both in terms of the number and strength of the detected significant correlations. This is likely due to the nature of the disease, which can affect different regions of the central nervous system and thus can potentially cause damage to motor functions indiscriminately in the upper or lower body districts.

A detailed examination of the magnitude of the relationships between upper and lower limb kinematic parameters showed that gait speed (and to a lesser extent, stride length) was the lower limb performance parameter that showed a higher number of significant correlations with upper limb variables. Moreover, larger correlations were found with hand-to-mouth velocity and adjusting sway. Changes in gait speed in pwMS, which represent one of the most distinctive features of the disease, are associated with a wide range of mobility and balance measures [30] and are mainly attributable to sensory changes and the resulting imbalance, muscle weakness or spasticity, and cerebellar ataxia [31]. It is likely that some of these factors may have a direct influence, albeit with different magnitudes, on both upper and lower limbs. For instance, one study showed that pwMS exhibit reduced isometric strength in upper limb muscles when compared to unaffected individuals [32] and often exhibit spasticity (although in a reduced percentage of cases with respect to that reported for lower limbs [33]). Thus, it appears reasonable to state that while both upper and lower limb motor impairments share a common root, which can be found in specific aspects of the disease, the expected impact on the respective functions may be quite different.

Similarly, it was interesting to observe that adjusting sway (i.e., the overall length of the fingertip trajectory during the phase dedicated to locating the mouth) was significantly correlated with most gait parameters. Adjusting sway is a measure of the adjustments necessary to reach the final position, and it can be considered representative of the degree of precision of the movement [23]. In this case, the detected moderate correlations with gait parameters are likely due to alterations in the way proprioceptive signals are processed, as they have consequences on postural control and thus, indirectly, even on gait.

Lastly, our data confirm the high degree of validity of the EDSS scale as predictor of walking performance, as demonstrated by the very high coefficients of correlation calculated for the investigated gait parameters. However, at the same time, it seems quite clear that such scale cannot be considered a reliable indicator of overall motor disability, since the correlations with upper limb kinematic parameters, although mostly significant, are largely reduced in magnitude. This is not surprising, considering the fact that ambulation represents the cardinal aspect on which the EDSS scale is based, but this finding also suggests a prudent approach to clinicians when they attempt to extrapolate overall motor disability estimation from EDSS data only.

Some limitations of the study should be acknowledged. Firstly, the sample here tested was limited, composed by pwMS affected by the relapsing-remitting form only, and quite heterogeneous in terms of disability level. Thus, further (and possibly longitudinal) tests are needed on a larger sample of pwMS with different forms of the disease (i.e., relapsing-remitting, primary, and secondary progressive MS) to clarify how the relationship between upper and lower limb motor abilities evolves during the disease course. Secondly, it would also be useful to investigate a wider range of upper limb functional tasks (i.e., reaching, grasping, and manipulation) to clarify whether the correlations reported here are task-specific or rather generalized. At last, as this was a monocentric study, caution should be taken with the generalization of its results.

## 5. Conclusions

This study investigated the existence of possible relationships between upper and lower limb functional impairments in pwMS who underwent a quantitative kinematic analysis of gait and hand-to-mouth movement. The results suggest that motor impairments of the two districts are partly correlated, particularly for gait speed (for lower limbs) and adjusting sway (for upper limbs), probably due to a specific set of MS features (specifically muscular weakness, spasticity, and proprioceptive deficits) that can influence both tasks, although in different ways. Since the EDSS scale does not seem to be suitable as an overall disability indicator, it may be necessary to perform specific tests to define upper limb impairments, and thus obtain a clearer detailed view of the alterations of functional motor tasks in MS.

## Figures and Tables

**Table 1:** Anthropometric and clinical features of the participants. Values are expressed as mean ± standard deviation.

	MS	HC	*p*-Value
Participants, # (M, F)	28 (14M, 14F)	21 (11M, 10F)	0.815
Age (years)	45.3 ± 9.7	49.6 ± 19.0	0.244
Body mass (kg)	64.0 ± 12.5	65.2 ± 11.2	0.622
Height (cm)	166.0 ± 9.3	167.5 ± 7.9	0.407
EDSS score	4.0 ± 1.8	—	—

MS: multiple sclerosis, HC: healthy controls, EDSS: Expanded Disability Status Scale.

**Table 2:** Comparison between kinematic parameters of gait (top) and hand-to-mouth task (bottom) for people with multiple sclerosis (MS) and healthy controls (HC). Values are expressed as mean ± standard deviation.

		MS	HC	*p*-Value
Lower limbs (gait)	Gait speed (m/s)	0.74 ± 0.35	1.28 ± 0.15	<0.001^*^
	Stride length (m)	0.94 ± 0.28	1.36 ± 0.12	<0.001^*^
	Cadence (steps/min)	90.90 ± 20.71	115.18 ± 8.03	<0.001^*^
	Step width (m)	0.22 ± 0.04	0.19 ± 0.03	0.001^*^

Upper limbs (hand-to-mouth)	Movement velocity	0.51 ± 0.12	0.58 ± 0.07	0.001^**^
	Going phase	45.84 ± 2.23	49.41 ± 3.27	<0.001^**^
	Adjusting phase	7.14 ± 4.09	3.72 ± 2.26	<0.001^**^
	Return phase	47.15 ± 3.79	44.38 ± 14.60	0.208
	Adjusting sway	3.81 ± 3.80	2.53 ± 1.95	0.053

The symbol ∗ denotes a significant difference in MS vs. HC after Bonferroni correction (*p* < 0.0125).

The symbol ∗∗ denotes a significant difference in MS vs. HC after Bonferroni correction (*p* < 0.01).

MS: multiple sclerosis; HC: healthy controls.

**Table 3:** Spearman's rho coefficients of correlation between upper and lower limb kinematic parameters in people with multiple sclerosis (MS) and healthy controls (HC).

Lower limb (gait)									
		Gait speed		Stride length		Cadence		Step width	
		MS	HC	MS	HC	MS	HC	MS	HC
Upper limb (hand-to-mouth)	Movement velocity	0.433^**^	0.337^*^	0.127	0.081	0.452^**^	0.071	0.075	−0.105
	Going phase	−0.069	−0.171	−0.317^*^	−0.191	−0.253	−0.146	0.374^**^	−0.042
	Adjusting phase	−0.274^*^	−0.363^*^	−0.122	−0.115	−0.108	−0.442^**^	−0.215	0.127
	Return phase	−0.370^**^	0.246	0.353^**^	0.072	0.315^*^	0.006	−0.079	−0.110
	Adjusting sway	−0.510^**^	−0.496^*^	−0.326^*^	−0.347^*^	−0.407^**^	−0.297	−0.115	−0.196

^*^ *p* < 0.05;

^**^ *p* < 0.01;

MS: multiple sclerosis; HC: healthy controls.

**Table 4:** Spearman's rho coefficients of correlation between EDSS score and upper and lower limb kinematic parameters in people with multiple sclerosis.

	Kinematic parameter		EDSS score
Lower limb (gait)	Gait speed (m/s)	vs.	−0.819^**^
	Stride length (m)		−0.810^**^
	Cadence (steps/min)		−0.745^**^
	Step width (m)		0.389^**^

Upper limb (hand-to-mouth)	Movement velocity	vs.	−0.353^**^
	Going phase		−0.427^**^
	Adjusting phase		0.422^**^
	Return phase		−0.169
	Adjusting sway		0.275^**^

^**^ *p* < 0.01;

EDSS: Expanded disability status scale.

## Data Availability

Data will be available upon request.
